# Network-level functional topological changes after mindfulness-based cognitive therapy in mood dysregulated adolescents at familial risk for bipolar disorder: a pilot study

**DOI:** 10.1186/s12888-021-03211-4

**Published:** 2021-04-28

**Authors:** Kun Qin, Du Lei, Jing Yang, Wenbin Li, Maxwell J. Tallman, Luis Rodrigo Patino Duran, Thomas J. Blom, Kaitlyn M. Bruns, Sian Cotton, John A. Sweeney, Qiyong Gong, Melissa P. DelBello

**Affiliations:** 1 Huaxi MR Research Center (HMRRC), Department of Radiology, West China Hospital of Sichuan University, Chengdu, China; 2 Department of Psychiatry and Behavioral Neuroscience, University of Cincinnati College of Medicine, Cincinnati, OH USA; 3 Department of Family and Community Medicine, University of Cincinnati College of Medicine, Cincinnati, OH USA; 4 Research Unit of Psychoradiology, Chinese Academy of Medical Sciences, Chengdu, China; 5 Functional and Molecular Imaging Key Laboratory of Sichuan Province, Chengdu, China

**Keywords:** Resting-state functional magnetic resonance imaging, Graph theory, Emotion regulation, Psychoradiology, Bipolar disorder, Familial risk, Mindfulness-based intervention

## Abstract

**Background:**

Given that psychopharmacological approaches routinely used to treat mood-related problems may result in adverse outcomes in mood dysregulated adolescents at familial risk for bipolar disorder (BD), Mindfulness-Based Cognitive Therapy for Children (MBCT-C) provides an alternative effective and safe option. However, little is known about the brain mechanisms of beneficial outcomes from this intervention. Herein, we aimed to investigate the network-level neurofunctional effects of MBCT-C in mood dysregulated adolescents.

**Methods:**

Ten mood dysregulated adolescents at familial risk for BD underwent a 12-week MBCT-C intervention. Resting-state functional magnetic resonance imaging (fMRI) was performed prior to and following MBCT-C. Topological metrics of three intrinsic functional networks (default mode network (DMN), fronto-parietal network (FPN) and cingulo-opercular network (CON)) were investigated respectively using graph theory analysis.

**Results:**

Following MBCT-C, mood dysregulated adolescents showed increased global efficiency and decreased characteristic path length within both CON and FPN. Enhanced functional connectivity strength of frontal and limbic areas were identified within the DMN and CON. Moreover, change in characteristic path length within the CON was suggested to be significantly related to change in the Emotion Regulation Checklist score.

**Conclusions:**

12-week MBCT-C treatment in mood dysregulated adolescents at familial risk for BD yield network-level neurofunctional effects within the FPN and CON, suggesting enhanced functional integration of the dual-network. Decreased characteristic path length of the CON may be associated with the improvement of emotion regulation following mindfulness training. However, current findings derived from small sample size should be interpreted with caution. Future randomized controlled trials including larger samples are critical to validate our findings.

The online version contains supplementary material available at 10.1186/s12888-021-03211-4.

## Background

Owing to the high familial heritability of bipolar disorder (BD), offspring of parents with BD are predisposed to mood dysregulation characterized by persistent emotional irritability without definite manic episodes, which can be a precursor for developing BD later in life [1, 2]. No specific interventions or treatments for mood dysregulated adolescents have established efficacy to date. Antidepressants, which are commonly used to treat depression and anxiety in adolescents, may accelerate the onset of mood instability and ultimately, mania or hypomania [3]. Mood stabilizers and antipsychotics may likewise yield adverse metabolic effects [4]. Therefore, alternative psychosocial treatment options for mood dysregulation in offspring of parents with BD are of interest for early intervention and prevention.

Mindfulness-based interventions have been recently shown to effectively improve a range of psychiatric conditions [5]. Mindfulness-based cognitive therapy (MBCT) is an evidence-based, manualized and systematic mindfulness-based intervention that combines traditional cognitive-behavior therapy with novel mindfulness theory in an integrated manner [6]. MBCT has also been adapted into a child version (Mindfulness-Based Cognitive Therapy for Children, MBCT-C) to provide a more specialized strategy towards pediatric patients [7]. By reinforcing the attention to thoughts, emotions, and body sensations, MBCT-C can improve the ability to manage with stress, anxiety, depression, pain, and other psychologically adverse conditions in youth [7, 8]. Studies of mood dysregulated youth have found enhanced emotion regulation ability with anxiety disorders after systematic MBCT-C treatment [9]. Neuroimaging studies have also demonstrated outcome-related changes after mindfulness-based interventions in both functional and structural brain features in regions (e.g., prefrontal cortex (PFC), anterior cingulate cortex (ACC), and insula) that exhibit abnormalities in individuals at familial risk for BD [10, 11]. These findings suggest that MBCT-C may be an effective intervention strategy for mood dysregulation in high-risk youth via impact on specific brain systems.

Default mode network (DMN), fronto-parietal network (FPN) and cingulo-opercular network (CON) are important resting-state functional networks that have been widely implicated in mindfulness-based interventions [12–14]. Specifically, the DMN has been associated with diverse complex self-referential processes [15], and the FPN and CON are responsible for top-down cognitive control [16]. All these processes involved in the triple networks are highly consistent with the key neural mechanisms in mindfulness (e.g. attentional control, body awareness, emotion regulation and change in self-perspective) [10]. Task-based functional magnetic resonance imaging (fMRI) studies have shown enhanced regional functional activation following mindfulness practice in the dorsolateral PFC, medial PFC, ACC and insula [10], which are hub regions of the DMN, FPN and CON. Moreover, previous studies have also found that individuals at high familial risk for BD exhibited functional abnormalities in these regions and networks [17–19]. MBCT-C may therefore enhance the function of these networks and further facilitate these processes to accomplish therapeutic effects on mood dysregulated adolescents at familial risk for BD.

Though some progress has been made in understanding the neurofunctional mechanisms of Mindfulness-based interventions, previous studies primarily focused on regional aspects of the brain rather than analyzing brain changes at the network-level. In recent years, emergence of the graph theory analysis allows a high-order exploration of brain network organization to move beyond regional findings, providing novel insights into human brain connectome alterations associated with psychiatric disorders [20, 21]. In the context of graph theory, complex brain networks can be abstracted as a simplified graph via the definition of nodes and edges, which enabled further topological analysis within the graph structure. For the brain functional network assessed by resting-state fMRI, nodes are usually defined as parcellations of brain regions, and edges refers to the coupling of functional activities between pair of nodes. An increasing number of studies have begun to apply network topological analysis to patients with BD as well as individuals at familial risk [22–25], characterizing abnormal organization within disrupted brain networks. Several pilot studies have also identified topological reorganization of both structural and functional networks following diverse psychotherapeutic treatments [26, 27], as well as mindfulness-based interventions [14].

Hence, the aim of our current study was to use graph theory analysis of resting-state fMRI data to investigate the impact of MBCT-C on relevant intrinsic functional brain networks in mood dysregulated adolescents at familial risk for BD. We hypothesized that MBCT-C in mood dysregulated adolescents would yield a clinically relevant network-level neurofunctional effect on topological properties of DMN, FPN and CON.

## Methods

### Participants

Study participants were recruited from the University of Cincinnati BD high-risk cohort and local community. Participants were included if they: (1) had at least one biological parent diagnosed with BD type-I using the Structured Clinical Interview for DSM-IV [28]; (2) were mood dysregulated prior to MBCT-C as determined by a Children’s Depression Rating Scale-Revised (CDRS-R) score > 28, Young Mania Rating Scale (YMRS) score > 12, or Emotion Regulation Checklist (ERC) score < 27 [29–31]; (3) were fluent in English; and (4) agreed to complete at least 75% of sessions. Exclusion criteria were as follows: (1) previously documented diagnosis of mental retardation or an Intelligence Quotient (IQ) < 70; (2) present and lifetime diagnosis of BD or other psychotic disorders; (3) previous participation in mindfulness-based treatment; (4) substance use disorder (except nicotine or caffeine) within the past 90 days; (5) active suicidal ideation, intent or plan within the past 30 days or a baseline CDRS-R suicide score > 3; (6) adjustment of concurrent psychotropic medication during the 30 days prior to screening or plan to adjust during the course of study; (7) initiation of psychotherapy treatment within 2 months prior to screening or plan to initiate psychotherapy during the course of study; (8) severe psychiatric symptoms requiring hospitalization; (9) mood symptoms resulting from acute intoxication, acute medical illness or withdrawal from drugs; or (10) presence of a neurological disorder, significant head trauma or unstable medical illness. Kiddie-Schedule for Affective Disorders and Schizophrenia (KSADS) [32] was performed or reviewed by two psychiatrists (M.P.D and L.R.P.D) with established diagnostic reliability (kappa value > 0.9) to identify present and lifetime psychopathology for exclusion criteria 2 and 4. Other exclusion criteria were conducted by one of the child and adolescent psychiatrists (M.P.D and L.R.P.D) study investigators. This study was approved by the University of Cincinnati Institutional Review Board, and written informed assent and consent was obtained from all participants and their parents, respectively.

### MBCT-C procedure and clinical assessments

A master’s level graduate student and a doctoral level psychologist, with training and experience in MBCT treatment and child clinical work, served as facilitators to conduct the MBCT-C protocol. Each participant was asked to attend 75-min group sessions each week for the 12-week MBCT-C intervention. Facilitators began each session with a sitting meditation, then reviewed the previous session and at home practices, taught a new mindfulness exercises (e.g. mindful breathing, mindful listening), read a relevant group poem or story, distributed informational handouts, and reviewed home practice for the upcoming week. The themes across the 12 sessions were: 1) Being on Automatic Pilot; 2) Being Mindful Is Simple, but It Is Not Easy; 3) Who Am I; 4) A Taste of Mindfulness; 5) Music to Our Ears; 6) Sound Expressions; 7) Practice Looking; 8) Strengthening the Muscle of Attention; 9) Touching the World With Mindfulness; 10) What the Nose Knows; 11) Life Is Not a Rehearsal; and 12) Living with Presence, Compassion and Awareness. As the MBCT-C manual is designed for 8–12 years children which does not fit the age range of our samples well, we made minor adaptations to the MBCT-C protocol manual to explain the concepts in developmentally appropriate language. Moreover, considering age-appropriate differences in language and teaching methods, we further divided the 10 subjects into two groups (younger group: 13–15 years old; older group: 15–17 years old). This adaptation has been reported in our previous publication and proved to work well [33]. Fidelity of the practice was supervised weekly by a licensed doctoral-level clinical expert with experience in conducting and publishing on MBCT for 33 years.

The ERC that evaluates emotional regulation ability consists of 24 items using a four-point Likert-like scale and was completed by parents. The ERC contains two subscales: Emotion Regulation and Emotion Lability/negativity, which provides evidence about different aspects (e.g., lability, flexibility, valence, intensity and management) of emotion regulation in children [30, 34] and has been used to examine the offspring at risk for mood disorders [35]. Though the ERC is originally developed for children, the age range of our overall “at-risk” cohort is closer to youth containing both children and adolescents. Given no current consensus on measurements assessing emotion regulation ability, we chose the ERC to keep consistency as in other youth studies [36, 37]. CDRS-R and YMRS were administered to assess the severity of depressive and manic symptoms, respectively. It is noted that the CDRS-R is originally developed for children aging 6–12 years, but it has been widely used in adolescents in both observational studies and intervention trials [38, 39]. Children’s Global Assessment Scale (CGAS) was also completed to characterize the overall level of children’s global functioning [40]. Clinical ratings for each participant were conducted with the same parent and by the same clinician rater throughout the study. Assessments were completed prior to the first MBCT-C session and following the last session.

### fMRI acquisition and preprocessing

Resting-state fMRI was performed on a 4.0 Tesla whole body MRI system (Varian Unity INOVA, Varian Inc., Palo Alto, CA) using a T2-weighted gradient-echo echoplanar imaging pulse sequence. All participants had resting-state fMRI performed prior to the first MBCT-C session and again following the last session. The standard scanning procedure required participants to stay awake with their eyes fixed on a white cross in the center of a black screen while trying to keep their mind clear. Parameters of the sequence were as follows: field of view, 256 × 256 mm; matrix, 64 × 64; voxel size, 4 × 4 × 4 mm^3^; 35 axial slices; repetition time, 3000 ms; echo time, 30 ms. The scan lasted for 10 continuous minutes, generating 200 volumes.

Standard preprocessing procedures were implemented using the Data Processing & Analysis for (Resting-State) Brain Imaging (DPABI) toolbox [41]. The first 10 volumes were eliminated from each scan to ensure homogeneity of the Blood-Oxygen-Level-Dependent (BOLD) signal. Then, slice-timing correction and six-parameter head motion correction were performed to reduce intravolume acquisition time mismatch and intervolume spatial displacement, respectively. Corrected images were normalized to a 3 × 3 × 3 mm standard EPI template and subsequently smoothed with an 8 mm full width at half maximum Gaussian kernel. Finally, several denoising methods were applied, including linear detrending, temporal band pass filter (0.01–0.08 Hz) and nuisance covariate regression (white matter signal, cerebrospinal fluid signal and Friston-24 head motion parameters). None of the included participants exhibited excessive head motion with movement > 3 mm or rotation > 3 degree).

### Graph theory analysis

Graph theory analysis was performed using the Gretna toolbox [42]. We first applied a functional Region of Interest (ROI) atlas (*N* = 160) proposed by Dosenbach et al. to define the nodes of the whole-brain network [43]. Next, edges were obtained by calculating the Pearson correlation coefficients of the mean time series between all possible pairs of nodes. Once the whole-brain network was constructed, DMN (*N* = 34), FPN (*N* = 21) and CON (*N* = 32) components were extracted from the whole-brain network according to the divisions of subnetworks [43]. To remove spurious functional edges, we applied a range of thresholds based on sparsity instead of a single value threshold. Sparsity denoted the ratio of the number of existing edges divided by the maximum possible number of edges. The lower bound was determined depending on the network scale (i.e., number of nodes) to ensure the minimum number of edges was larger than N/2 × ln(N) [44], providing estimable network topological metrics. The upper bound was set to an empirical value of 0.4 to reach a reasonable sparsity trade-off for network analysis [45, 46]. Hence, the generated sparsity thresholds of DMN, FPN and CON ranged from 0.11 to 0.4, 0.16 to 0.4 and 0.12 to 0.4 with an interval of 0.01, respectively. This approach ultimately generated undirected and weighted networks at each sparsity threshold, which were used in the following estimation of topological metrics. Of note, we performed graph theory analysis on subnetworks instead of whole-brain networks to provide further insight into mesoscale network organization. From the methodological perspective, subnetworks can exactly be delineated as smaller but identical graph structure compared with whole-brain networks, enabling the topological analysis in conventional whole-brain manner. There have been substantial studies applying network-restricted graph theory analysis for intrinsic functional subnetworks [47–49], and the robustness of resulting topological metrics have also been confirmed [47]. On the other hand, subnetworks consist of group of brain regions that are known to be related to specific cognitive/behavioral functions [50, 51]. Considering the mechanism involved in mindfulness-based interventions, investigation on the DMN, FPN and CON provides more specificity for elucidating and interpreting neurofunctional alterations related to treatment effects.

Identical topological metrics were assessed in DMN, FPN and CON, respectively (for detailed definition and calculation of topological metrics, see Supplementary Information). Specifically, seven network topological metrics included network efficiency (E_net_), local efficiency (E_loc_), characteristic path length (L_p_), clustering coefficient (C_p_), normalized clustering coefficient (γ), normalized characteristic path length (λ) and small-worldness (σ), representing the network topological architecture [44, 52]. Three nodal topological metrics of all brain regions within the subnetworks including degree, efficiency and betweenness were calculated to evaluate the regional topological centralities [53–55]. To reach an explicit expression, we calculated the area under the curve (AUC) of topological metrics over all the sparsity thresholds instead of evaluating independent values based on each threshold (for detailed AUC calculation, see Figure S1 in Supplementary Information). The approach for AUC calculation in graph theory analysis has been widely used [56, 57], which can normalize the networks and make metrics less sensitive to threshold selection as well as head motion issues [58, 59]. Finally, we applied the network-based statistic (NBS) approach to identify alterations of edges in the targeted networks [60]. Briefly, NBS can identify altered functional connectivity prior to and following MBCT-C, and localize the connected components showing significant changes within DMN, CON and FPN.

### Statistical analysis

Considering the non-normality of data distributions for topological metrics, non-parametric permutation testing was used to analyze AUC values of topological metrics to identify significant differences between baseline and endpoint. We adopted a significance level of *p* < 0.05 for network topological metrics and used the False Discovery Rate (FDR) correction for multiple comparisons to maintain a corrected significance level of *p* < 0.05 for nodal topological metrics. If significant differences in topological metrics were found, AUC values of these metrics for each participant were extracted for subsequent correlation analysis. Partial correlation analysis was performed between change in topological metrics and change in clinical variables (ERC and CGAS). Age and sex were treated as covariates, and Bonferroni correction was applied to set a significance level of *p* < 0.05/N (N, number of correlations) for multiple correlation analysis.

For the NBS method, paired two-tailed t-tests were performed for all the edges irrespective of increased or decreased functional connectivity. The initial significance level was set at an uncorrected *p* < 0.001 to preliminarily gathered a set of altered edges. Within these altered edges, any connected components were subsequently identified using a breadth first search, and the size of identified components (i.e., number of links) was stored. To further determine the significance of connected components and solve the multiple comparison problem, a non-parametric permutation method (10,000 permutations) was used to estimate the significance of each component according to their size (number of edges). For each permutation, we repeated paired t-tests under the same threshold (*p* < 0.001) with random exchange of pre-treatment and post-treatment data and identified the maximal connected component size at the same time. The connected component size derived from the actual comparison would be significant if its size ranked top 5% of the 10,000 maximal component sizes derived from permutations (*p* < 0.05, family-wise error corrected).

## Results

### Demographic and clinical characteristics

Ten mood dysregulated adolescents with an average age of 14.6 years (age range: 13–17 years) participated in a 12-week MBCT-C clinical trial. Sixty percent of them were girls, 80% were Caucasian, and 20% were African American. Contrary to our hypothesis of improvement in clinical measures, no significant changes in CDRS-R (*p* = 0.474), YMRS (*p* = 0.553), CGAS (*p* = 0.059) and ERC (*p* = 0.102) scores were observed following MBCT-C treatment. Detailed demographic and clinical information are summarized in Table 1.

**Table 1:** Demographic and clinical characteristics of included mood dysregulated youth at familial risk for bipolar disorder

Variables	Youth (*n* = 10)		*p* value ^a^
	Baseline	Endpoint	
Age (years)	14.6 ± 1.8	–	N/A
Sex (male/female)	4/6	–	N/A
Race (Caucasian/African American)	8/2	–	N/A
CDRS-R	34.0 ± 17.4	29.7 ± 8.9	0.474
YMRS	5.4 ± 3.6	5.1 ± 3.4	0.553
CGAS	69.7 ± 10.8	78.0 ± 11.7	0.059
ERC	65.0 ± 10.9	69.6 ± 15.8	0.102

Quantitative data are presented as mean ± standard deviation

^a^ All *p* values were obtained by paired-samples Wilcoxon signed rank test

*Abbreviations*: *N/A* not applicable, *CDRS-R* children’s depression rating scale-revised, *YMRS* young manic rating scale, *CGAS* children’s global assessment scale, *ERC* emotion regulation checklist

### Changes in network and nodal topological metrics after MBCT-C

**Fig. 1:**
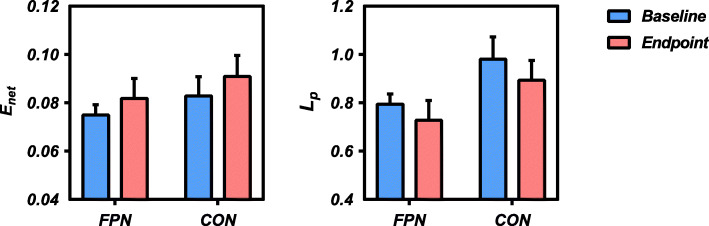
Significant changes in network topological metrics of the FPN and CON before and after MBCT-C. *Abbreviations*: FPN, fronto-parietal network; CON, cingulo-opercular network; MBCT-C, Mindfulness-Based Cognitive Therapy for Children; E_net_, network efficiency; L_p_, characteristic path length

Mood dysregulated adolescents showed significantly higher network efficiency (FPN *p* = 0.014, CON *p* = 0.020) and lower characteristic path length (FPN *p* = 0.017, CON *p* = 0.023) within the FPN and CON following MBCT-C (Fig. 1). No significant alterations of any network topological metrics within the DMN were observed. Nodal topological analysis of nodal metrics did not identify any regional changes within the DMN, FPN or CON that survived FDR correction.

### Changes in functional connectivity after MBCT-C

**Fig. 2:**
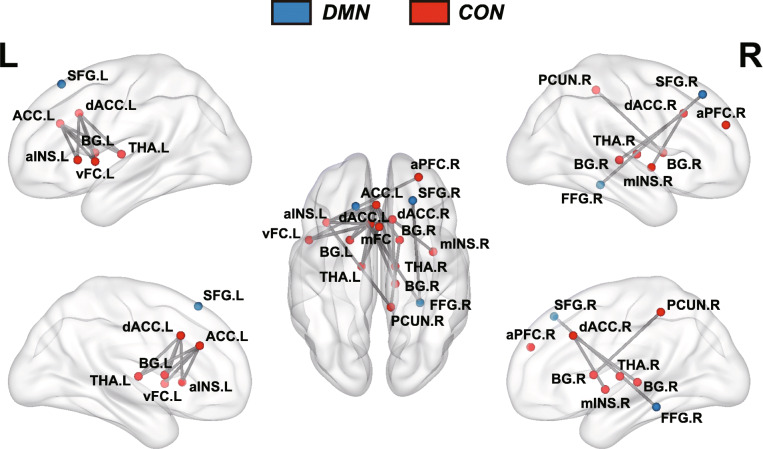
Brain map showing components of the DMN and CON with increased functional connectivity following MBCT-C. Nodes denote brain regions and edges denote functional connectivities. Nodes in DMN were shown in bule, while nodes in CON were shown in red. *Abbreviations*: DMN, default mode network; CON, cingulo-opercular network; MBCT-C, Mindfulness-Based Cognitive Therapy for Children; ACC, anterior cingulate cortex; dACC, dorsal anterior cingulate cortex; aINS, anterior insula; mINS, middle insula; mFC, medial frontal cortex; vFC, ventral frontal cortex; aPFC, anterior prefrontal cortex; PCUN, precuneus; SFG, superior frontal gyrus; FFG, fusiform gryus; THA, thalamus; BG, basal ganglia; L, left; R, right

NBS analysis revealed a connected component within the CON comprising 14 nodes and 19 connections with increased functional connectivity in mood dysregulated adolescents following MBCT-C (Fig. 2). Nodes within the component were mainly involved in the ACC, dorsolateral PFC, basal ganglia, insula and thalamus. For the DMN, we also localized a component with increased functional connectivity in mood dysregulated adolescents following MBCT-C. The DMN component had 3 nodes and 2 connections, which included the bilateral superior frontal gyrus and right fusiform. No connectivity changes within the FPN were detected.

### Relationship between topological metrics and clinical variables

**Fig. 3:**
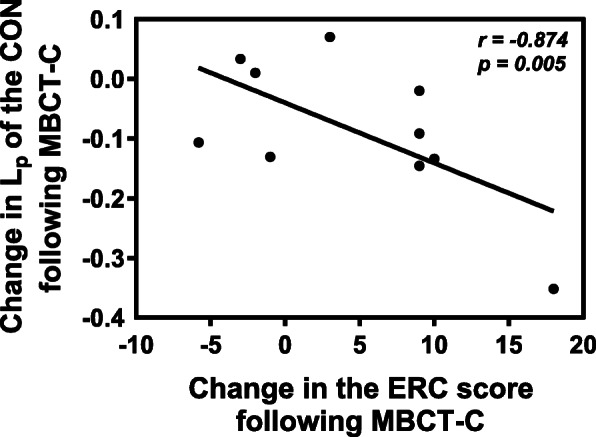
Relationship between change in characteristic path length of the CON and change in the ERC score. Change here was shown as “endpoint values – baseline values”. *Abbreviations*: L_p_, characteristic path length; ERC, Emotion Regulation Checklist; MBCT-C, Mindfulness-Based Cognitive Therapy for Children; CON, cingulo-opercular network

Change in the ERC score was significantly associated with change in characteristic path length of the CON (Fig. 3). There was no significant correlation between changes in the clinical variables and changes in network topological metrics of the FPN.

## Discussion

The current study applied a network-restricted graph theory analysis to investigate neurofunctional effects of MBCT-C in a pilot group of adolescents at risk for BD with mood dysregulation. Given the involvement of mindfulness in self-referential and cognitive control functions (e.g., attentional control, emotion regulation, and self-awareness) [61], three resting-state intrinsic functional networks including the DMN, FPN and CON were examined to explore the neural basis of MBCT-C. We found the FPN and CON both exhibited an increased network efficiency and a decreased characteristic path length in mood dysregulated adolescents at familial risk for BD following MBCT-C. Altered functional connectivity patterns mainly involving in frontal and limbic regions were identified via the NBS analysis. Furthermore, changes in characteristic path length of the CON was associated with change in the ERC score following MBCT-C. Although we failed to observe a statistically significant improvement in any clinical measures, these salient network-level findings that were related to clinical changes in the clinically non-significant context may provide interesting insights into neurofunctional basis of MBCT-C.

Human brain network architecture conforms to the small-world prototype with superiority of information processing and communication assessed by network topological metrics [62, 63]. In general, network efficiency and characteristic path length are thought to represent aspects of functional integration, while local efficiency and clustering coefficient are involved in functional segregation [55]. Regarding both FPN and CON, we found increased network efficiency and decreased characteristic path length following MBCT-C, suggesting an improved functional integration on specific brain systems related to top-down cognitive control processes. However, we failed to identify similar enhancement of functional integration in the DMN, which are known to be associated with the self-referential processes and mindfulness [12]. We speculated that this negative finding might be related to our specific participants. It has been well established that excessive self-referential processes are usually involved in internalizing disorders [64, 65], such as major depressive disorder and anxiety disorder. As for individuals with BD or at familial risk for BD, they suffer less abnormal self-referential processes or ruminative symptoms. Mindfulness-related therapeutic effects may therefore mainly focus on emotion regulation processes, which lead to significant alterations in topological organizations of the cognitive control dual-network system.

Although the FPN and CON are both involved in top-down cognitive control, their respective roles are different. The CON shows sustained activity across trials within a cognitive task to maintain the awareness of task-related goals and the stability of goal-directed behaviors during sustained cognitive activity, while the FPN contributes to cognitive control in briefer discrete epochs in a trial-by-trial way, feeding back error information and adjusting behavior planning in real time [16, 66]. We identified a relationship between improved functional integration and increase in ERC scores within the CON, but similar association was not observed for the FPN, suggesting that improved emotion regulation after MBCT-C was more directly related to the CON that supports sustained context-relevant cognitive control. From another perspective, hub regions of the CON (e.g., insula) are strongly associated with emotion [67], while hub regions of the FPN (e.G. *inferior* parietal lobule) are widely implicated in attentional control [61] and known to be densely connected with other attention-related regions [66]. For novices at mindfulness practice, frontal and parietal regions are usually activated to escalate the attention level and to obtain a deeper state of meditation [68]. Therefore, topological changes in the FPN may potentially suggest the enhanced attentional control during MBCT-C, assisting in achieving therapeutic benefits on emotion regulation from mindfulness practice.

At the connectivity level, altered functional connectivity within the DMN and CON following MBCT-C was observed. We found that increased functional connectivity was mainly involved in the frontal and limbic regions. Consistent with our findings, previous task-based fMRI evidence from studies of BD offspring with anxiety showed increases in activity of the ACC and insula when participants were presented emotional stimuli following 12-weeks MBCT-C treatment [69], suggesting a functional recruitment of limbic regions following mindfulness-based treatment. Moreover, disruption of the fronto-limbic system has been identified in the abnormal regulation of emotion in diverse psychiatric conditions [69–72]. Therefore, enhanced functional coupling of fronto-limbic system following MBCT-C may be related to the process of mindful emotion regulation. From the clinical perspective, we failed to find significant improvement in any clinical measures following MBCT-C, which might be ascribed to the small sample size. Moreover, the inconsistency between neuroimaging and clinical findings may also implicate reduced or more variable changes in behavioral ratings that we failed to capture, or perhaps a greater sensitivity of brain imaging data for detecting positive changes. After the MBCT-C intervention, it is also possible that brain function changes precede later-evolving behavioral changes, which means that mindfulness-related neurofunctional changes mediate the longer-term clinical improvement.

Limitations of the current study need to be considered. First, the lack of a control group makes it difficult to reliably interpret the identified brain changes as intervention-related findings. These changes may also reflect longitudinal changes that would have happened without intervention, as well as changes related to brain development or pathophysiological changes related to mood dysregulation as a possible prodromal marker of BD development. Though these brain functional changes were consistent with findings reported in previous mindfulness studies, conclusion from the current flawed design should be treated with caution. Second, in this pilot study, only 10 adolescents were recruited. While our findings are promising, the small sample size may limit reproducibility and the statistical power to detect more subtle changes in brain function after treatment. Fourth, our treatment was not blinded, which may bias the study toward positive effects via high expectancy effects. Future studies with larger samples, well-designed control group and longer-term follow-up are needed to further investigate the neural basis of positive clinical changes after mindfulness training.

## Conclusions

Our current pilot study is the first to examine neurofunctional effects of MBCT-C on mood dysregulated adolescents at network-level. Convergent findings on the dual-network showed improved functional integration of both FPN and CON in mood dysregulated adolescents at familial risk for BD following MBCT-C. We also identified connected components with increased functional connectivity within the DMN and CON, mainly comprising frontal and limbic regions. Moreover, improved network integration of the CON was associated with altered emotion regulation ability following MBCT-C. These findings suggest that network topological changes within the CON and FPN following MBCT-C may represent important and distinct neurofunctional effects of mindfulness on the dual-network in mood dysregulated BD offspring. Owing to small sample size in the current studies, results and interpretations are heuristic. Future studies with large cohort and control group should be performed to replicate and refine the current findings.

## Supplementary Information

**Additional file 1.**

## Data Availability

The datasets used in the current study are available from the corresponding author on reasonable request.

## Abbreviations

MBCT-C: Mindfulness-based cognitive therapy for children
BD: Bipolar disorder
fMRI: Functional magnetic resonance imaging
DMN: Default mode network
FPN: Fronto-parietal network
CON: Cingulo-opercular network
PFC: Prefrontal cortex
ACC: Anterior cingulate cortex
CDRS-R: Children’s depression rating scale-revised
YMRS: Young mania rating scale
ERC: Emotion regulation checklist
CGAS: Children’s global assessment scale
AUC: Area under the curve
NBS: Network-based statistic
FDR: False discovery rate
