# Supplementary material for: Network-level functional topological changes after mindfulness-based cognitive therapy in mood dysregulated adolescents at familial risk for bipolar disorder: a pilot study
Source: BMC Psychiatry. 2021 Apr 28;21:213. doi: 10.1186/s12888-021-03211-4 (PMC8080341; doi:10.1186/s12888-021-03211-4)

**Online Supplementary Materials**

**Network-level functional topological changes after Mindfulness-Based Cognitive Therapy in mood dysregulated adolescents at familial risk for bipolar disorder: A pilot study**

Kun Qin^1#^, Du Lei^2#^, Jing Yang^1^, Wenbin Li^1,2^, Maxwell J. Tallman^2^, Luis Rodrigo Patino Duran^2^, Thomas J. Blom^2^, Kaitlyn M. Bruns^2^, Sian Cotton^3^, John A. Sweeney^1,2^, Qiyong Gong^1,4*^, Melissa P. DelBello^2^

^#^ Kun Qin and Du Lei contributed to this work equally.

**Affiliations:**

^1^ Huaxi MR Research Center (HMRRC), Department of Radiology, West China Hospital of Sichuan University, Chengdu, China.

^2^ Department of Psychiatry and Behavioral Neuroscience, University of Cincinnati College of Medicine, Cincinnati, Ohio, USA.

^3^ Department of Family and Community Medicine, University of Cincinnati College of Medicine, Cincinnati, Ohio, USA.

^4^ Psychoradiology Research Unit of Chinese Academy of Medical Sciences, Functional and Molecular Imaging Key Laboratory of Sichuan Province, West China Hospital of Sichuan University, Chengdu, China.

***Corresponding Author:**

Dr. Qiyong Gong can be contacted at Huaxi MR Research Center (HMRRC), Department of Radiology, West China Hospital, Sichuan University, No.37 Guo Xue Xiang, 610041 Chengdu, China. E-mail: qiyonggong@hmrrc.org.cn

**Table of contents**

1. Network Topological metrics

1.1 Network efficiency

1.2 Local efficiency

1.3 Characteristic path length

1,4 Clustering coefficient

1.5 Normalized Characteristic path length

1,6 Normalized Clustering coefficient

1.7 small-worldness

2. Nodal Topological metrics

2.1 Nodal degree

2.2 Nodal efficiency

2.3 Nodal betweenness

*Figure S1* Calculation of AUC for topological metrics

1. **Network topological metrics**

*1.1 Network efficiency* ${(E}_{glob})$ evaluates the efficiency of whole-network information exchange. For a graph G with N nodes and M edges, network efficiency is defined as:

$$E_{net}(G)=\frac{1}{N\left( N-1 \right)}\sum_{a\neq b\in G} \frac{1}{d_{ab}}$$

where $d_{ab}$ is the shortest weighted path between node $a$ and $b$.

*1.2 Local efficiency* $(E_{loc})$ measures the average efficiency of subgraphs across nodes:

$$E_{loc}(G)=\frac{1}{N}\sum_{a\in G} E_{glob}(G_{a})$$

where $G_{a}$ is the subgraph consisting of index node $a$ and its neighbors.

*1.3 Characteristic path length* ${(L}_{p})$ is similar to network efficiency and equivalent to its inverse:

$$L_{p}(G)=\frac{1}{N}\sum_{a\in G} L_{p}(G_{a})$$

$$L_{p}(G_{a})=\frac{\sum_{b\in G, b\neq a} d_{ab}}{N-1}$$

where $G_{a}$ is the subgraph consisting of index node a and its neighbors, $L_{p}(G_{a})$ denotes the average shortest weighted path between node $a$ and all the other nodes.

*1.4 Clustering coefficient* ${(C}_{p})$ measures the possibility of one node’s neighbors that are also mutually connected, which reflects the local segregation together with local efficiency:

$$C_{p}(G)=\frac{1}{N}\sum_{a\in G} C_{p}(G_{a})$$

$$C_{p}(G_{a})=\frac{\sum_{b,c\in G} \sqrt[3]{w_{ab}w_{ac}w_{bc}}}{k_{a}(k_{a}-1)}$$

where $G_{a}$ is the subgraph consisting of index node a and its neighbors, $C_{p}(G_{a})$ denotes the average weighted clustering coefficient of node $a$, $k_{a}$ is the degree of node $a$ and $w$ is the connection weights between a pair of nodes.

*1.5 Normalized characteristic path length* ($\lambda$) is determined by scaling real characteristic path length with mean characteristic path length derived from random networks. Herein, random networks were generated for 100 times for robustness:

$$\lambda(G)=\frac{L_{p}(G_{real})}{L_{p}(G_{random})}$$

*1.6 Normalized clustering coefficient* $(\gamma)$ is determined by scaling real clustering coefficient with mean clustering coefficient derived from random networks. Herein, random networks were generated for 100 times for robustness:

$$\gamma(G)=\frac{C_{p}(G_{real})}{C_{p}(G_{random})}$$

*1.7 Small-worldness* ($\sigma$) is defined as the ratio of $\gamma$ and $\lambda$:

$$\sigma(G)=\frac{\gamma(G)}{\lambda(G)}$$

1. **Nodal topological metrics**

*2.1 Nodal degree* measures the connection of the index node with all the other nodes in a graph:

$$k_{a}=\sum_{a\in G,a\neq b} w_{ab}$$

where $w_{ab}$ denotes the weighted connection between node $a$ and $b$.

*2.2 Nodal efficiency* is defined as the ability of information flow of a given node:

$$e_{a}=\frac{1}{N-1}\sum_{a\in G,a\neq b} \frac{1}{d_{ab}}$$

where $d_{ab}$ is the shortest weighted path between node $a$ and $b$.

*2.3 Nodal betweenness* is determined to assess the influence of a given node on information flow in the graph:

$$b_{a}=\frac{1}{(N-1)(N-2)}\sum_{a\neq b\neq c\in G} \frac{\theta_{bc}(a)}{\theta_{bc}}$$

where $\theta_{bc}$ is the total number of shortest paths between node $b$ and $c$, and $\theta_{bc}(a)$ is the number of those shortest paths that path through node $a$.

**Figure S1** Calculation of area under the curve (AUC) for topological metrics. Take small-worldness ($\sigma$) for example, the AUC was calculated over all the sparsity thresholds ranging from $S_{min}$ to $S_{max}$ with interval of $\Delta s$ as $\sigma=\sum\frac{\Delta s}{2}[\sigma\left( s_{i} \right)+\sigma\left( s_{i+1} \right)]$.
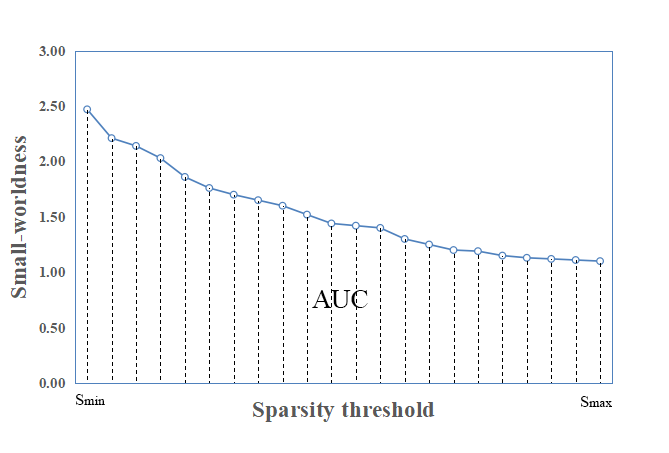

Supplement: Supplementary file 1 — Additional file 1. [file 12888_2021_3211_MOESM1_ESM.docx]
